# Belize It: Early Resident Experience in a Global Surgery Rotation

**DOI:** 10.7759/cureus.8096

**Published:** 2020-05-13

**Authors:** Nandita Rao, John Vance, David Walters, Bracken Burns

**Affiliations:** 1 Surgery, East Tennessee State University, Johnson City, USA; 2 General Surgery, Bristol Surgical Associates, Bristol, USA; 3 Surgery, Quillen College of Medicine, East Tennessee State University, Johnson City, USA

**Keywords:** global surgery, international surgery rotations, international programs, surgery rotations

## Abstract

A global surgical rotation program has been developed at La Loma Luz Adventist Hospital in San Ignacio, Belize, by the Department of Surgery at East Tennessee State University in 2014. It encompasses a one-month rotation for surgery residents to travel to Belize, accompanied by a senior surgical attending, to participate in direct patient care. Residents are able to operate under supervision and practice both perioperative and medical management. Practitioners often collaborate with permanent facility surgeons and internists in order to assist with cases, discuss different practice strategies, and, ultimately, tailor patient care. In addition to providing residents with surgical experience, this rotation aims to advance the overall standard of medical care available to the community. Additional aims include healthcare promotion and education of patients. While this rotation was developed to provide residents surgical experience in an underserved country, we hope that it will further cultivate volunteerism and foster future participation.

## Introduction

The small developing country of Belize lies on the northeastern side of Central America and covers approximately 23,000 square miles (roughly the size of Massachusetts) [1]. Since its relatively recent independence in 1981, it has been battling multiple issues related to healthcare access and promotion of preventative medicine. According to the Central Intelligence Agency, in 2008 and 2012, there were 0.83 physicians and 1.1 hospital beds allotted per 1,000 members of the population [1]. Most practicing physicians have received international training and, there has been a growing international effort to help fill deficits in the medical field. With a population of more than 350,000, the most common etiologies of adult death are ischemic heart disease, diabetes-related complications, and interpersonal violence [2].

There are 11 registered hospitals (8 public and 3 private) in Belize. The largest available hospital is Karl Heusner Memorial Hospital (KHMH) located in Belize City [3]. KHMH offers the highest level of medical care in the country, serving a client base of around 100,000 [4]. The remaining areas of healthcare access include 60 known public clinics, which are distributed in both urban and rural settings. La Loma Luz (LLL) is one of the private institutions, approximately two hours driving distance from Belize City, which offers 20 inpatient beds and services from departments including internal medicine, surgery, obstetrics/gynecology, pediatrics, urology, and radiology. The hospital was constructed and has been maintained through faith-based charity organizations and facilitates around 40-50 patient visits on a daily basis [4]. Global surgery rotation at East Tennessee State University (ETSU) was introduced in 2014, with plans to contribute to resident global surgical education by way of bi-yearly month-long rotations.

The purpose of this study is to describe early trends in resident patient care involvement after the establishment of a global surgical rotation.

## Materials and methods

Residents who had completed junior years of training (preferably first and second post-graduate years) were eligible to apply, and a single resident accompanied a senior surgical attending for a duration of four weeks. ETSU’s general surgery department was able to provide a standard resident salary with travel, health, and evacuation insurance. Housing was set up at a faculty member guesthouse, accompanied by a nighttime safety guard, with food supplied by LLL. General working hours were from 8:00 a.m. to 7:00 p.m., during which time residents were typically in the operating room for scheduled procedures, emergency department for admissions, and inpatient wards for consultations. As there is only one exclusive general surgeon permanently employed by the hospital, in addition to traveling specialists (i.e. urologists, ENTs, obstetrician-gynecologists), residents also provide assistance to other specialized cases. Over the course of the first two and a quarter years, ETSU has provided global surgery training to a total of five surgical residents. Data collected by these residents included case logs of patient demographics and procedures performed.

## Results

**Figure 1:**
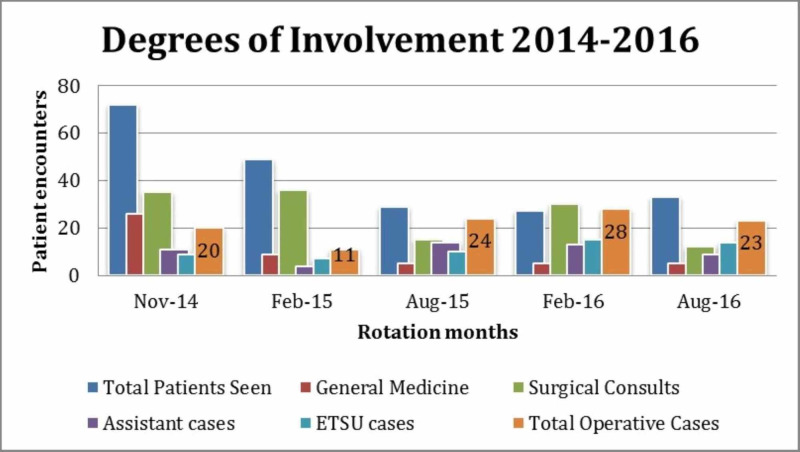
Review of resident involvement during rotation months ETSU, East Tennessee State University

During the past two and a quarter years, totaling five separate rotation months, participation in patient care has been cataloged into degrees of involvement and types of cases encountered. These data have been tracked to observe trends in resident involvement and diversity of cases encountered. Figure 1 outlines documented patient cases ranging between types of resident involvement since program initiation. This illustrates that overall medical care has transitioned from predominantly primary care/medical medicine to surgery-related interventions.

**Figure 2:**
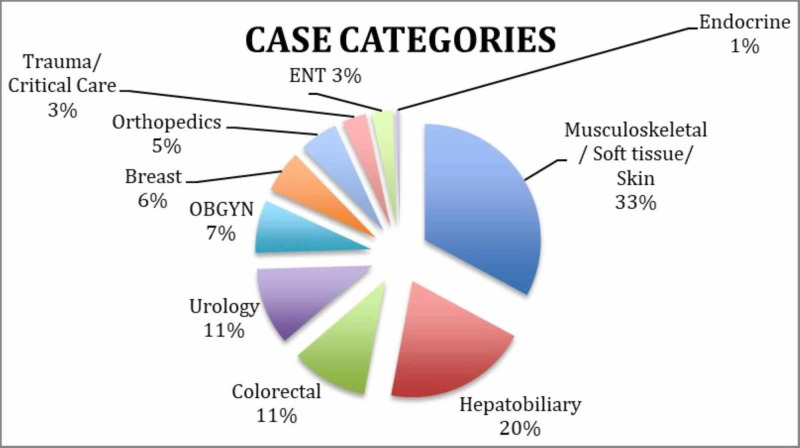
Categorization of surgical cases over the three most recent rotations (months included: August 2015, February 2016, and August 2016)

Figure 2 is a further categorization of interventions recorded with direct operative participation. It classifies surgeries based on data using data from the three most recent ETSU rotations. The most common procedure overall was laparoscopic cholecystectomy under the hepatobiliary system (totaling 36 cases); however, the most common system involved was musculoskeletal (26 total herniorrhaphies and 28 total skin/soft tissue excisions).

In addition to independent operative cases, residents were able to provide training to on-site physicians regarding the basic principles of laparoscopy. Most cases at LLL are performed in a traditional open technique due to a lack of necessary equipment and trained staff. Four different faculty mentors from ETSU volunteered their time and expertise specifically in general, trauma, and vascular surgery. Procedures ranged in complexity from prolonged critical care management of severe industrial related injuries to very simple yet necessary interventions such as arterial or urinary catheter insertion when no other trained healthcare workers were present.

## Discussion

Looking back at Figure 1, it is possible that the transition from predominantly primary care/medicine interactions to surgical procedures may be secondary to progressive familiarity of local residents to the surgical capabilities of the visiting team. As the ability to obtain additional surgical resources became available, it was easier to expand the surgical capability during these rotations. Furthermore, the data in Figure 2 can be used to reflect the potential needs of the local population as the cases performed were on uninsured or underinsured individuals. Barriers to furthering medical care at LLL and globally include internal factors such as insufficient quantity and training of staff, financial support for resources, and physical availability to accommodate patients, and external factors such as patient transportation and time constraints [5]. Many important factors in selecting a site for an international surgical rotation include community resources and needs, safety, educational materials, food, and lodging. Rotations must be optional for trainees and of a minimum of two weeks in length to create enough time for a valuable experience. Teams willing to travel internationally also need to be dedicated to initiating and sustaining partnerships. Valuable data obtained from subsequent visits will help further elicit risk factors for predominant diseases and, in turn, promote research efforts in preventative medicine. A large barrier in this ongoing project is the lack of available data for analysis. Most hospital logs are maintained on paper charts and therefore difficult to obtain off-site, lacking details of the patient background and circumstances of the operation. Furthermore, despite development of hospital facilities, efforts are less impactful without improvement in patient access to care. If resources necessary for certain operations or physicians with expertise are not available, patients will have to travel to a larger facility for high levels of medical care.

Future plans to expand the program are underway. While providing general surgical coverage at the facility, ETSU is also developing cross-departmental collaborative partnerships. The ETSU School of Public Health is constructing an international rotation that will collaborate with the residency program in order to better understand outcomes of involvement in the Belize health system. Additionally, this will allow for potential research projects to be developed. International surgical rotations are becoming increasingly important and are becoming more prevalent due to globalization and the wide array of benefits [6]. The potential benefit of international surgical rotations for surgery residents is to provide experience functioning with limited resources and increased exposure to an increased breadth of organ system pathologies (i.e., urology, orthopedics, obstetrician-gynecologist) [7]. It also provides access to necessary healthcare for those less-developed countries that are burdened by disease and lack resources [8]. In addition to providing a unique surgical rotation for the surgical residents at ETSU, hopefully this rotation could benefit the citizens of San Ignacio, Belize, who seek care at LLL. By studying the underserved patients seen on this rotation, it may help predict common diseases in the community. This can be further compared with other populations in the country and potentially help identify risk factors or predictors of morbidity and mortality.

In addition to the resident learning opportunities on this rotation, there are also opportunities for the native surgeons of Belize. With the experience of the senior surgical attending and the availability of resources garnered for this surgical rotation, Belize physicians are able to expand upon their current capabilities. The increase in surgical capability allows the native physicians to provide and expand the scope of care outside of the surgical rotation. By enhancing the scope of procedures that native physicians feel comfortable performing, this can impact care year-round.

## Conclusions

The future goal of this program is not only to provide a surgical resident experience and short term medical care but also to eventually develop the overall medical community. This will take both progressive effort and continued re-evaluation and required adjustments. This community-based effort could expand resources and improve external limiting factors such as easier access of patients to healthcare. The partnership between LLL and ETSU is one of many possible models for international engagement and surgical education. We hope that our early resident experience in a global surgical rotation will provide information about the trends we encountered after establishing our rotation that may be useful to others who may be establishing or wish to establish similar training experiences.
